# Human DNA-dependent protein kinase activation mechanism

**DOI:** 10.1038/s41594-022-00881-w

**Published:** 2023-01-05

**Authors:** Shikang Liang, Tom L. Blundell

**Affiliations:** 1 Department of Biochemistry, University of Cambridge, Cambridge, UK; 2 Present Address: Heart and Lung Research Institute, Department of Medicine, University of Cambridge, Royal Papworth Hospital NHS Foundation Trust, Cambridge, UK

**Keywords:** Non-homologous-end joining, Kinases, Cryoelectron microscopy, DNA damage response, Enzyme mechanisms

## Abstract

DNA-dependent protein kinase (DNA-PK), a multicomponent complex including the DNA-PK catalytic subunit and Ku70/80 heterodimer together with DNA, is central to human DNA damage response and repair. Using a DNA-PK-selective inhibitor (M3814), we identified from one dataset two cryo-EM structures of the human DNA-PK complex in different states, the intermediate state and the active state. Here we show that activation of the kinase is regulated through conformational changes caused by the binding ligand and the string region (residues 802–846) of the DNA-PK catalytic subunit, particularly the helix-hairpin-helix motif (residues 816–836) that interacts with DNA. These observations demonstrate the regulatory role of the ligand and explain why DNA-PK is DNA dependent. Cooperation and coordination among binding partners, disordered flexible regions and mechanically flexible HEAT repeats modulate the activation of the kinase. Together with previous findings, these results provide a better molecular understanding of DNA-PK catalysis.

## Abstract (web summary)

Cryo-EM structures of human DNA-dependent protein kinase in intermediate and active states reveal the molecular mechanism of the allosteric activation of the atypical kinase complex, explaining why it is DNA dependent.

## Main

The DNA-PK of vertebrate cells is central to the DNA damage response and the nonhomologous end-joining (NHEJ) pathway for the repair of DNA double-strand breaks, the most dangerous form of DNA damage, which may lead to genomic instability, carcinogenesis and cell death^1,2^. DNA-PK was first identified when addition of double-stranded DNA (dsDNA) to cell extracts was observed to stimulate the phosphorylation of certain proteins. The catalytic activity was found to be related to a very large polypeptide, now known as the DNA-PK catalytic subunit (DNA-PKcs), with 4128 amino acid residues and a molecular weight of ~470 kDa^3–5^. Later, it was discovered that the effective recruitment and activation of DNA-PKcs by DNA requires the heterodimer Ku70/80, which is formed by two subunits of molecular weights approximately 70 kDa and 80 kDa^6,7^. The kinase domain of DNA-PKcs was found to be highly similar to that of phosphoinositide 3-kinase, belonging to the family of phosphoinositide 3-kinase-related kinases (PIKKs), which includes ATM, ATR, mTOR, SMG1 and TRRAP^8^. PIKK family members have the greatest similarity in the kinase domain, which is surrounded by a conserved α-helical FAT (FRAP, ATM and TRAPP) domain together with the PIKK regulatory domain (PRD) and the FATC domain. All PIKKs also have an α-solenoid amino-terminal region, which consists mainly of HEAT repeats that have important regulatory roles.

There have been structural studies on both DNA-PKcs and the DNA-PK holoenzyme by several independent groups^9–18^. DNA-PKcs has been described as comprising three structural units, the N-terminal unit (1–892), the circular cradle (893–2801) and the head (2802–4128), where the kinase region is located between residues 3565 and 4100 (refs. ^9,10^). The assembly of the DNA-PK holoenzyme and its higher-order dimerization in NHEJ complexes have also been described^11–17^. However, the molecular mechanism of DNA-PK activation remains unclear, with gaps between ligand-bound DNA-PKcs and ligand-bound intermediate or active DNA-PK^13,18^.

Our previous work suggested that inhibitors have a similar mode of structural regulation to that of ATP binding DNA-PKcs^18^. Using the latest-generation DNA-PK selective inhibitor M3814 to mimic ATP binding, while at the same time slowing down and inhibiting kinase catalysis, we succeeded in obtaining two different conformations of the DNA-PK–ligand complex from one cryo-EM dataset: the intermediate state, where the inhibitor binds to the ATP pocket before the closure of the kinase domain, and the active state, where the kinase domain is closed up and the ligand is brought next to the substrate-binding site. Together with the results of previous work on DNA-PKcs, DNA-PK and DNA-PKcs–ligand complexes, these findings provide structural insight into the progression from ligand binding through the full catalytic pathway^13,14,18^.

## Results and discussion

### Structure overview

**Fig. 1:**
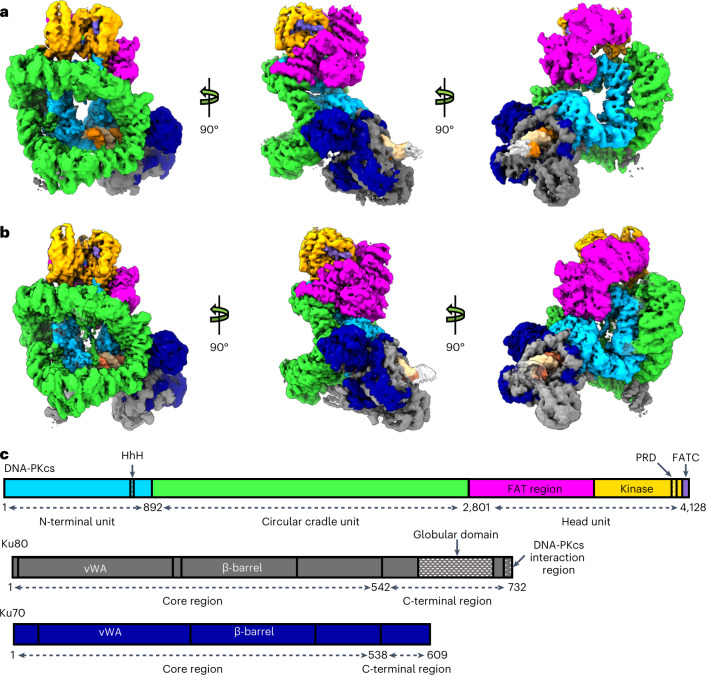
Overview of DNA-PK from structures defined by cryo-EM. **a**, Front (left), side (middle) and back (right) views of the Coulomb potential map of DNA-PK in the intermediate state. **b**, Front (left), side (middle) and back (right) views of the Coulomb potential map of DNA-PK in the active state. **c**, Schematic representation of sequences of DNA-PKcs, Ku80 and Ku70 with structural regions of DNA-PKcs colored differently: the N-terminal unit is in blue, the circular cradle unit in green, the FAT region of the head unit in magenta, the kinase of the head unit in yellow and the FATC of the head unit in purple; Ku80 is shown in gray and Ku70 in navy.

**Extended Data Fig. 1:**
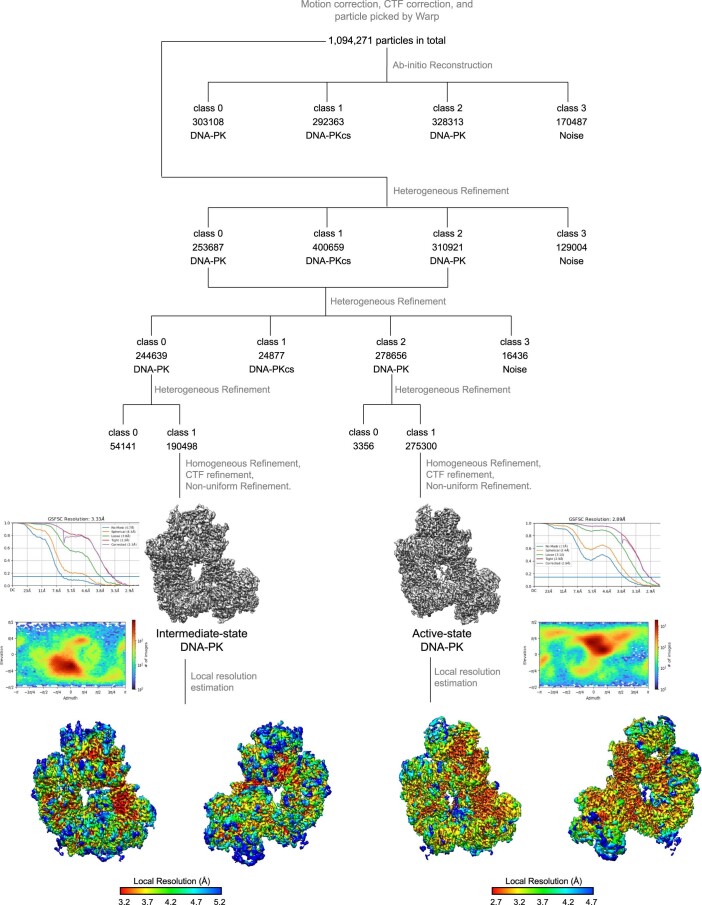
Cryo-EM data processing of the DNA-PK/M3814 complex.

DNA-PKcs natively purified from HeLa cell nuclear extract was incubated on ice for 1 h with purified recombinant Ku70/80, DNA and an excess of DNA-PK selective inhibitor M3814. The dataset was collected on a Gatan K3 detector and processed (Extended Data Fig. 1 and Table 1). From the dataset, we identified an intermediate state of DNA-PK, allowing definition at 3.2 Å resolution following density modification with ResolveCryoEM, before closure of the kinase domain and catalysis (Fig. 1a). Moreover, we managed to stabilize and lock the active DNA-PK in the transient conformation adopted for catalysis with a closed kinase domain, giving a cryo-EM model at a resolution of 2.8 Å following density modification with ResolveCryoEM (Fig. 1b), in which the inhibitor occupies a position next to the substrate-binding site.

**Table 1:** Cryo-EM data collection, refinement and validation statistics

	Intermediate DNA-PK (EMDB-14546) (PDB 7Z88)	Active DNA-PK (EMDB-14545) (PDB 7Z87)
**Data collection and processing**		
Magnification	130k	130k
Voltage (kV)	300	300
Electron exposure (e^–^/Å^2^)	47.22	47.22
Defocus range (μm)	−1.1 to −2.3	−1.1 to −2.3
Pixel size (Å)	0.652	0.652
Symmetry imposed	C1	C1
Initial particle images (no.)	1094271	1094271
Final particle images (no.)	190498	275300
Map resolution (Å)	3.33	2.89
FSC threshold	0.143	0.143
Map resolution range (Å)	2.9 to >10	2.7 to >10
**Refinement**		
Initial model used (PDB code)	6ZHA, 7K1N	6ZHA, 7K0Y
Model resolution (Å)	3.33	2.89
FSC threshold	0.143	0.143
Model resolution range (Å)	n/a	n/a
Map sharpening *B* factor (Å^2^)	75.2	78.3
Model composition		
Nonhydrogen atoms	38754	39708
Protein residues	4750	4840
Ligands	1	1
*B* factors (Å^2^)		
Protein	24	126
Ligand	16	101
R.m.s. deviations		
Bond lengths (Å)	0.005	0.006
Bond angles (°)	0.909	0.842
Validation		
MolProbity score	2.4	2.3
Clashscore	21	16
Poor rotamers (%)	0.17	0.13
Ramachandran plot		
Favored (%)	88.62	89.39
Allowed (%)	11.29	10.48
Disallowed (%)	0.09	0.13

### Cryo-EM structure of DNA-PK in the intermediate state

**Fig. 2:**
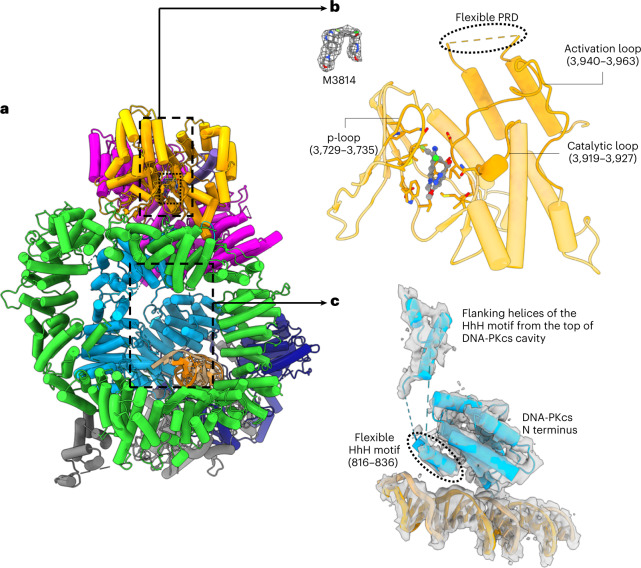
The structure of DNA-PK in the intermediate state defined by cryo-EM. **a**, DNA-PK in intermediate state. **b**, Close-up view of the kinase catalytic core around the DNA-PK selective inhibitor (M3814). The corresponding Coulomb potential map of the inhibitor and the two-dimensional structure are shown in the upper left panel. The inhibitor sits in the ATP-binding groove, coordinated by the p-loop (3729–3735), catalytic loop (3919–3927) and activation loop (3940–3963). The PRD becomes flexible in the intermediate state of DNA-PK, opening the substrate-binding site for catalysis. **c**, Close-up view of the N-terminal region around the HhH motif (816–836) and the corresponding Coulomb potential map. In the intermediate structure of DNA-PK, the HhH motif position is not fixed between the DNA end and N terminus but is connected through a highly flexible linking peptide to the top of the DNA-PKcs central cavity, as indicated in **a**. The color scheme is consistent with that of Fig. 1.

The inhibitor docks in the center of the ATP-binding groove in the intermediate-state DNA-PK (Fig. 2a,b). The overall assembly of the kinase complex is similar to those previously defined^12–14,17^. The DNA–Ku70/80 core complex interacts with the N-terminal unit and circular cradle of DNA-PKcs, and the DNA passes under the N-terminal unit, while the carboxy-terminal region of Ku80 docks on the circular cradle. However, there are many differences in the conformations and structural details.

DNA-PK in the intermediate state has several structured regions that are flexible. In Ku80, these include the vWA domain (6–242), the six-helical bundle (595–706) and the 12 residues (721–732) of the C-terminal region (543–732)^19,20^. Within the DNA-PKcs component of DNA-PK, there are several structured regions that are flexible, including the N-terminal region, close to where the DNA end enters, and a part of the kinase region within the head unit, especially the four-helical FKBP12-rapamycin-binding (FRB) domain (3582–3675)^21^. Another interesting flexible region in DNA-PKcs is the helix-hairpin-helix (HhH) motif between the N terminus of DNA-PKcs and the DNA end (Fig. 2c), which is likely to come from a flexible region (802–846) hanging down in the DNA-PKcs central cavity formed by the circular cradle and N-terminal unit. It is clear that in the higher-resolution active-state DNA-PK structure, the HhH motif (816–836) is essential not only for allosteric activation and conformational transition but also for stabilizing the interaction between the DNA-PKcs N-terminal region and the head unit.

**Extended Data Fig. 2:**
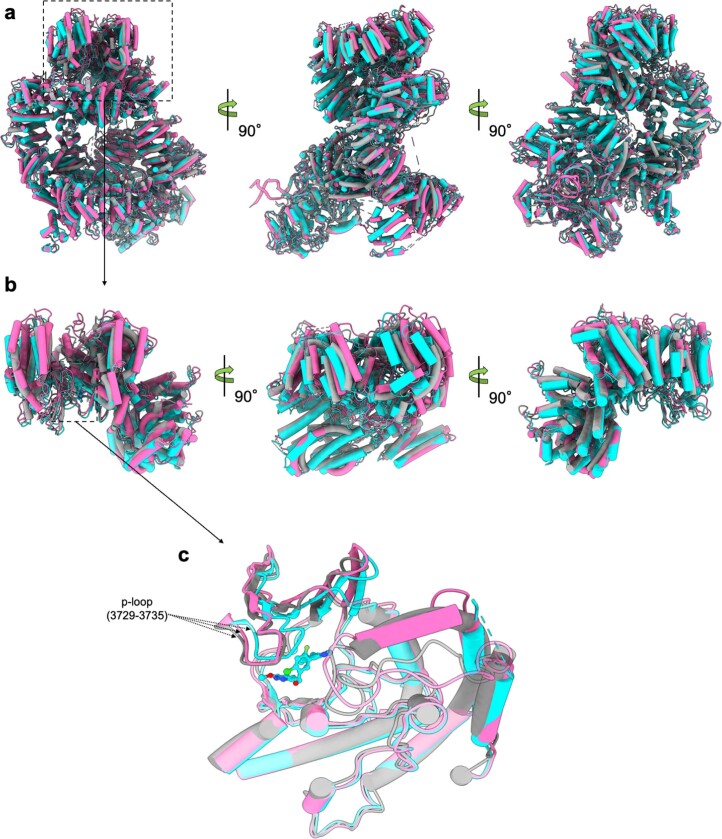
Comparison of DNA-PK in the intermediate state with inactive DNA-PKs. **a**, Superimposed DNA-PKs in the intermediate state (in cyan) and apo DNA-PKs (PDB code: 6ZHA in grey; PDB code: 7K1N in pink) when aligned by the DNA ends, including front (left), side (middle) and back (right) views. The main conformational differences lie in the Head unit of DNA-PKcs. **b**, Front (left), side (middle) and back (right) views of the superimposed Head units in the intermediate state and apo state when align by the DNA ends. Helices of the Head unit of the inactive apo DNA-PKs mostly cluster together while helices of the intermediate DNA-PK tend to move toward the N terminus and DNA end. **c**, Magnified view of the superimposed kinase region in intermediate state and apo state when align by the C-lobe of the kinase. The addition of the inhibitor changes the conformation of N-lobe via interacting with the p-loop, leading to parallel movement of the p-loop flanking sheets.

Compared with the previously published structures of the inactive-state apo-DNA-PK, the major conformational differences lie in the head unit of DNA-PKcs (Extended Data Fig. 2a,b)^13,14^. In the intermediate-state complex, the head unit moves towards the DNA end and the N-terminal unit of DNA-PKcs (Extended Data Fig. 2a,b). When the ligand binds, the p-loop moves close to it (Extended Data Fig. 2c). This leads to concerted movements involving conformational changes of the flanking β-sheets of the p-loop and movement in the hydrophobic core of the head unit, resulting in overall conformational changes. More importantly, the outward movement of the head unit releases the PRD (4009–4039) from its position in the apo kinase (Fig. 2b and Extended Data Fig. 2c). In the intermediate complex, there is only weak signal near the PRD position, which indicates that the PRD is destabilized. When the blockage of PRD is removed, the kinase active site becomes available for substrate peptide binding and subsequent catalytic activity.

### Cryo-EM structure of DNA-PK in the active state

**Fig. 3:**
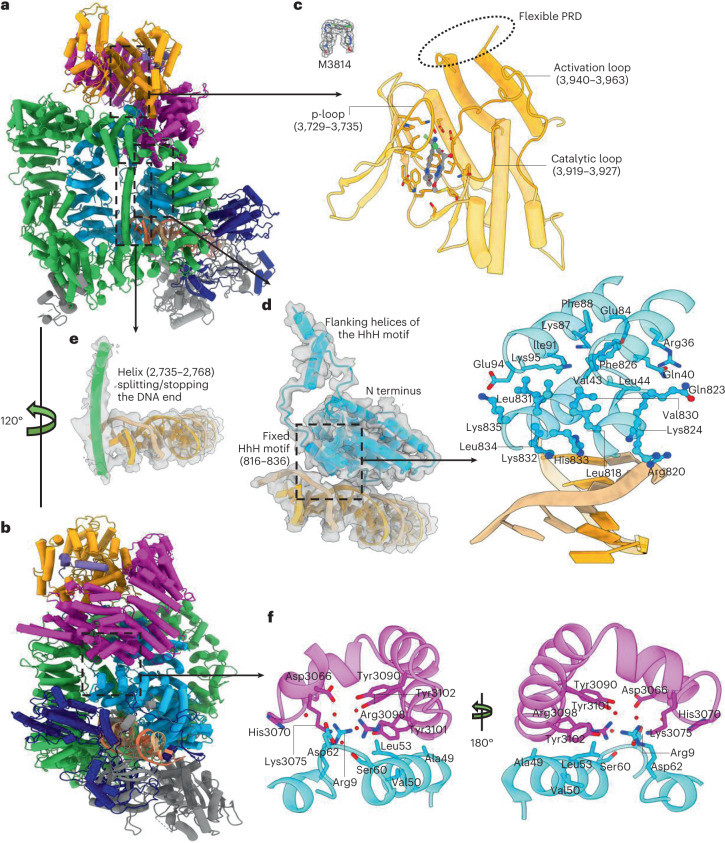
The structure of DNA-PK in the active state defined by cryo-EM. **a**,**b**, Two views, related by a rotation of 120^o^, of DNA-PK in the active state. **c**, Close-up view of the kinase catalytic core around the inhibitor. The corresponding Coulomb potential map of the inhibitor is shown in the upper left of this panel. **d**, The DNA end and N-terminal region around the HhH motif (816–836), the corresponding Coulomb potential map and a closer view of the interaction interfaces. In active DNA-PK, the HhH motif is fixed between the DNA end and N terminus. The peptide string connecting HhH motif to the top of the DNA-PKcs central cavity is restricted and can be clearly traced in the map. **e**, DNA end with the DNA-end-binding helix (2735–2768) and the corresponding Coulomb potential map. **f**, Interaction interface between DNA-PKcs N terminus and the FAT region. Color scheme is consistent with that of Fig. 1.

In the active state of DNA-PK, the kinase core is closed and the ligand M3814 is moved close to the substrate-binding site (Fig. 3a–c). Similar to the intermediate-state kinase complex, some structured regions are flexible in the active DNA-PK structure. Most parts of Ku80 remain flexible, including the vWA domain, the six-helical bundle (595–706) and the 12 C-terminal residues (721–732). However, in DNA-PKcs, the previously flexible N-terminal region and HhH motif (816–836) are now stabilized between the FAT region and the DNA terminus (Fig. 3d).

On one side of the HhH motif, there are electrostatic interactions between positively charged residues, including Arg820, Lys824, Lys832 and His833, and the negatively charged DNA phosphate backbone. On the other side of the HhH motif, hydrophobic interactions define the binding with the DNA-PKcs N-terminal region. Phe826 of the HhH motif docks into the groove between the two helices that pack onto the HhH motif, interacting with the side chains of Phe88 and Ile91 and the main chain atoms of Arg36, Gln40, Glu84 and Lys87. The side chains of Leu818, Val830, Leu831 and Leu834 also interact with the hydrophobic surface composed of the side chains of Val43, Leu44 and Ile91 and the main chain atoms of Glu94 and Lys95. In addition to the hydrophobic interaction, there is an electrostatic interaction between Lys835 and Glu94 and hydrogen bonds among Gln823, Arg36 and Gln40 (Fig. 3d).

The position of the N-terminal region is also defined by interactions with the FAT region of the head unit. Tyr3101 lies in the hydrophobic pocket formed by Ala49, Val50 and Leu53. There are water-mediated side chain interactions among the polar and charged residues of the interface, including Asp3066, His3070, Lys3075, Tyr3090, Arg3098 and Tyr3102 from the FAT region and Arg9, Ser60 and Asp62 from the N-terminal unit (Fig. 3f). Furthermore, in the active form of DNA-PK, a DNA-end-binding helix (2735–2768) from the flexible region of DNA-PKcs lies near the ABCDE cluster in the middle of the DNA-PKcs central cavity, restricting inward movement of the DNA (Fig. 3e). The stabilization of the DNA-end-binding helix is likely to be caused by the fixation of the activation HhH motif together with its flanking peptide string and the DNA end.

**Extended Data Fig. 3:**
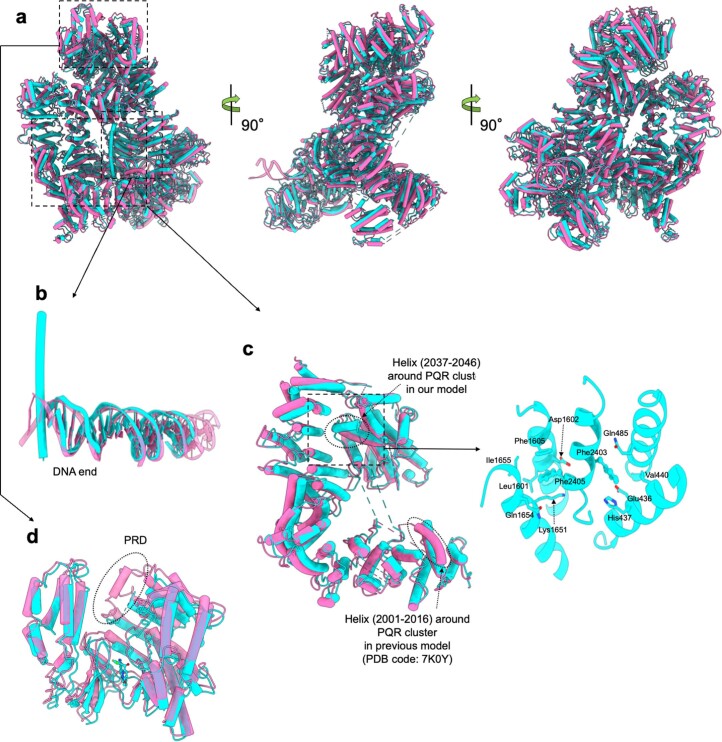
Comparison of DNA-PK in the active state with another activated DNA-PK model. **a**, Superimposed DNA-PK of our model in the active state (in cyan) and the previously published DNA-PK (PDB code: 7K0Y in pink), including front (left), side (middle) and back (right) views. There are global conformational differences. **b**, Magnified view of superimposed DNA ends of the two models. In our model, the DNA end does not go in as far due to the DNA stopping helix. **c**, Magnified view of the superimposed region of Circular Cradle and N-terminal unit near the helix around PQR cluster (2037–2046) in the two models. In our model, the helix sits in between the Circular Cradle and N-terminal unit. In the previous model, the helix (2001–2016) around PQR cluster sits on the Circular Cradle. **d**, Magnified view of the superimposed Head units in our model and the previously described apo DNA-PK close to the active state. In our model, the addition of ligand on ATP binding groove destabilizes or releases the PRD from its position in inactive state and the Head unit is further pulled down to the DNA end. The whole Head unit is also further pulled down towards the DNA end.

Compared with the previously proposed model of an activated DNA-PK, the structure of the active holoenzyme, described here, is more compact, showing different conformational changes (Extended Data Fig. 3a)^13^. The DNA end lies in the center of the holoenzyme and to different extents all regions of the holoenzyme move towards it (Extended Data Fig. 3a). Moreover, the lengths of the DNA strands coming into the DNA-PKcs central cavity are different (Extended Data Fig. 3b). A further difference is that the helix from the region around the PQR cluster continues to dock in the cleft between the N-terminal unit and the circular cradle in our active form, similar to the intermediate DNA-PK (Extended Data Fig. 3c). In addition, the PRD, instead of having a 115° outward rotation, is destabilized and removed from the substrate-binding site (Extended Data Fig. 3d), although different, destabilization and rotation have the same effect in making the substrate-binding site available for substrates to bind.

### Intermediate-to-active-state transition of DNA-PK

**Fig. 4:**
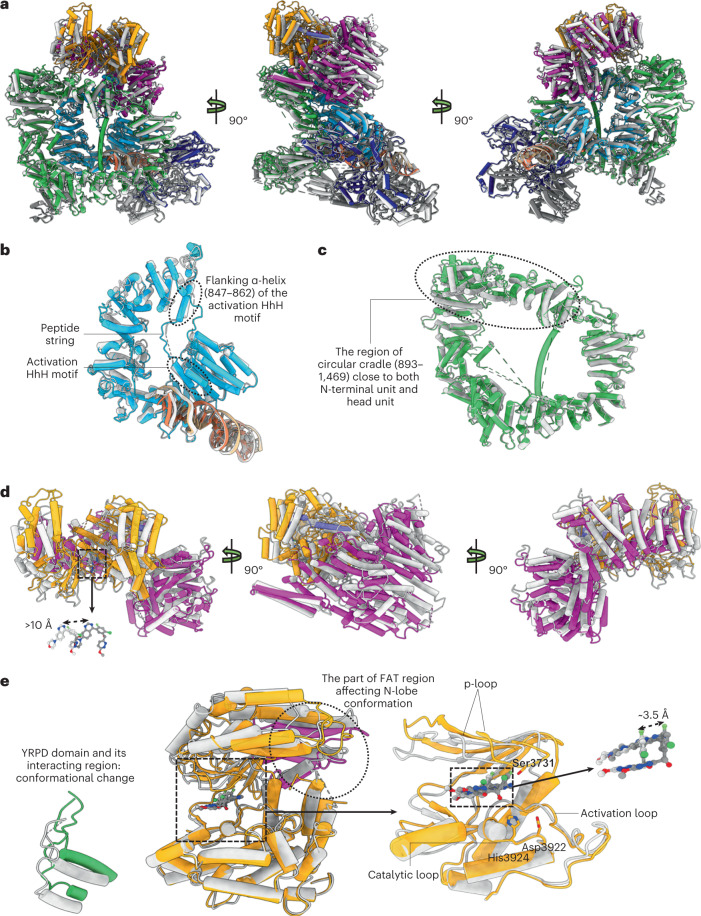
Transition of DNA-PK from the intermediate state to the active state. **a**, Front (left), side (middle) and back (right) views of superimposed DNA-PK molecules in the intermediate state and the active state when aligned by DNA ends. The color scheme previously used for DNA-PK in the active state is retained, whereas DNA-PK in the intermediate state is shown in silver. **b**, Close-up view of the superimposed N-terminal units and DNA ends in the intermediate state and active state when aligned by DNA ends. **c**, Magnified view of the superimposed circular cradle units in the intermediate state and the active state when aligned by DNA ends. **d**, Front (left), side (middle) and back (right) views of superimposed head units in the intermediate state and the active state when aligned by DNA ends. Close-up views of the superimposed DNA-PK selective inhibitors in the intermediate state and the active state when aligned by DNA ends, showing that the ligand is moved more than 10 Å during the transition. **e**, Close-up views of the superimposed kinase region, YRPD and its interacting region in the intermediate state and active state when aligned by the C-lobe of the kinase. YRPD and its interacting region become closer to the kinase catalytic site. The N-lobe moves towards the C-lobe during the activation, facilitated by the movement of the neighboring FAT region (3490–3565). Relative to the kinase C-lobe, the ligand is moved around 3.5 Å closer to the catalytic site during activation.

The holoenzyme undergoes a series of conformational changes during the transition from the intermediate to the active state (Fig. 4a). Most changes lie in DNA-PKcs, especially in the N-terminal and head units and the part of the circular cradle unit that is close to them (893–1469) (Fig. 4a–d).

According to our structures, the main trigger for kinase activation and transition is the fixation of the HhH motif (816–836) (Fig. 4b). Stabilization of the activation HhH motif between the DNA terminus and the N-terminal region induces a conformational change of the latter. Furthermore, it restricts the conformation of the previously disordered flexible peptide (837–846) linking to the following α-helix (847–862) from the top of the DNA-PKcs central cavity (Fig. 4b). The peptide string pulls the connected helix closer to the DNA end and leads to further serial downward movements of the nearby FAT region, which comes in contact with the N-terminal region. The conformational changes are also passed on to the whole molecule, especially to the kinase region (Fig. 4a–d). The peptide string acts as a ‘pull switch’ for activating the kinase, and the inhibitor in the active state is moved more than 10 Å away from its position in the intermediate state (Fig. 4d).

To understand how the kinase region is closed during activation, the kinase regions of DNA-PK in the two states were aligned by superimposing the C-lobes of the two kinase structures (Fig. 4e). This demonstrates a concerted movement of the N-lobe and neighboring FAT region (3490–3565), pushing the p-loop towards the C-lobe. The inhibitor molecule moves about 3.5 Å from its previous position relative to the C-lobe (Fig. 4e) towards the catalytic loop that contains the catalytic aspartate (Asp 3922) and the neighboring positively charged His 3924, which helps to localize the γ-phosphate of ATP together with Ser 3731 from the p-loop (Fig. 4e).

Previously, it was proposed that YRPD (2775–2795) and its interacting region (2569–2585) are important in regulation of the ABCDE cluster and autophosphorylation^22,23^. During the transition from the intermediate state to the active state, YRPD domain and its interacting region move closer to the catalytic site, indicating the *cis*-autophosphorylation of the ABCDE domain (Fig. 4e).

### Structural mechanism of DNA-PK activation

The structural studies of DNA-PKcs and DNA-PK, together with the intermediate and active DNA-PK, now provide a better understanding of the concerted movements of different domains during allosteric activation.

**Fig. 5:**
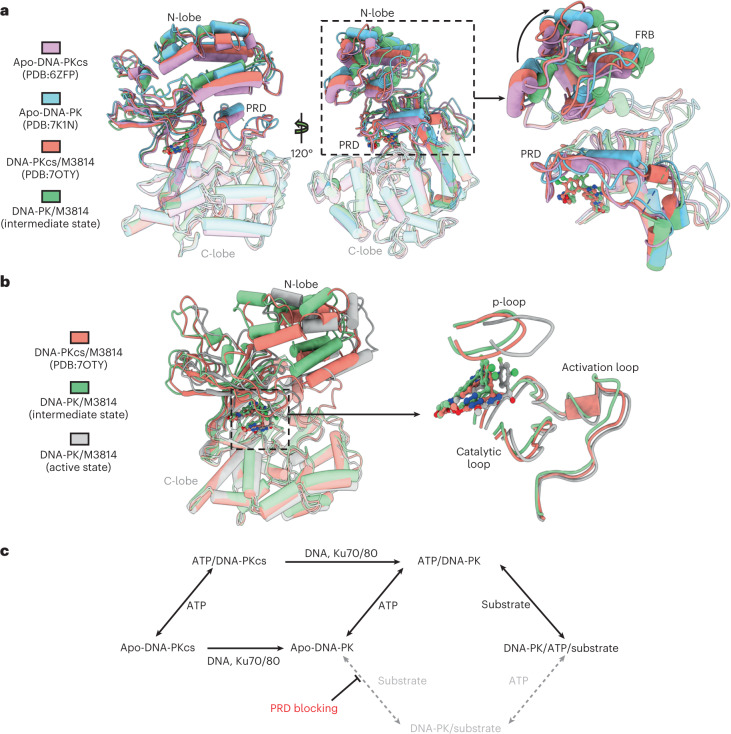
DNA-PK activation mechanism. **a**, Different views of superimposed kinase domains of DNA-PKcs and DNA-PK models when aligned by the C-lobe of the kinase. Kinase domains of DNA-PKcs from different apo models, including apo-DNA-PKcs (PDB: 6ZFP), apo-DNA-PK (PDB: 7K1N) and DNA-PKcs/M3814 (PDB: 7OTY)^13,14,18^, are superimposed on the one of intermediate DNA-PK. **b**, Superimposed kinase domains of DNA-PKcs and DNA-PK models during DNA-PK activation progression and close-up view of the ligand movement. **c**, Schematic diagram of the DNA-PK catalytic cycle.

Here, we focus on comparison of the kinase domains of different DNA-PKcs-related models, aligned by the C-lobe. Comparison of apo-DNA-PKcs and apo-DNA-PK indicates that the assembly of DNA-PK holoenzyme itself leads to an opening of the N-lobe, involving outward moving of the FRB domain and partial relaxation of the PRD (Fig. 5a)^13,14^. When a ligand binds to the ATP-binding site in DNA-PKcs, the PRD tilts but remains docked on the substrate-binding site^18^. With binding of the inhibitor to the ATP pocket in DNA-PK, the FRB domain further opens compared with that in apo-DNA-PKcs and apo-DNA-PK, leading to the release of the PRD (Fig. 5a).

Moreover, we can now better understand the progression from DNA-PKcs to DNA-PK in the catalytic cycle. The assembly of DNA-PK from ligand-bound DNA-PKcs or ligand binding to apo-DNA-PK affect the conformation of the head unit, open the FRB domain and release PRD from the substrate-binding site. Stabilization of the activation HhH motif between the DNA end and the N-terminal bridge induces global conformational changes within the holoenzyme complex, including N-lobe movement and closure of the kinase domain to move the ligand towards the substrate-binding site for catalysis (Fig. 5b). Of note, considering the high concentration of ATP in the cell, the in vivo structural progression of DNA-PK activation should start from ligand-bound DNA-PKcs and proceed to intermediate and active DNA-PK. In general, DNA, Ku70/80 and ligand binding, together with the activation HhH motif far from the DNA-PKcs head region, allosterically activate the kinase. Similar concerted movements among HEAT repeats and the kinase domain have previously been visualized in other PIKK members, and it is tempting to hypothesize that the cooperation among binding partners, the disordered flexible region and the structurally plastic HEAT repeats regulate functions of PIKK family^24^.

Among the phosphorylation targets of DNA-PK, self-phosphorylation is probably the most studied^25–28^. According to our active kinase model, the ABCDE cluster (2609–2647) is on a flexible region of polypeptide, which could have access to the kinase catalytic site and therefore could be phosphorylated in *cis*. The movement of the YRPD domain and its interacting region also suggests autophosphorylation of the ABCDE cluster in a *cis* manner. However, the PQR cluster (2023–2056) of DNA-PKcs can only be autophosphorylated in *trans* owing to its distance from the kinase catalytic region. Studies of NHEJ complexes containing DNA-PK have revealed the assembly of different dimers that probably mediate autophosphorylation in *trans*^14–16^. In these models, the Ku80 C-terminal region and other NHEJ components including XLF, XRCC4 and DNA ligase IV appear to assemble as a scaffold. A recent publication has revealed an interesting new conformation of DNA-PK after self-phosphorylation on the ABCDE cluster that is in line with the previous SAX model on the displacement of the N-terminal HEAT domain, as the N-terminal HEAT domain opens greatly^17,29^. Of note, in the autophosphorylated DNA-PK in complex with Artemis, the activation HhH motif cannot be stabilized by interactions between the DNA end and N-terminal arm owing to the Artemis blockage and remains in a destabilization state^17^. Therefore, the kinase domain is not closed, and DNA-PK is in an inactive state halting further phosphorylation before end processing and removal of the Artemis catalytic domain. However, how the phosphorylation on the flexible ABCDE domain affects the overall conformation of the HEAT repeats, especially the N-terminal region, remains unclear and requires further investigation.

Nevertheless, with high-resolution DNA-PK structures in different states, our findings reveal the structural mechanism of allosteric activation of the atypical kinase DNA-PK, the key step of its catalytic cycle, and offer improved models for future structure-guided drug discovery of new cancer therapeutics targeting the DNA-PK holoenzyme.

## Methods

### Purification of Ku70/80 and DNA-PKcs

Purifications of Ku70/80 and DNA-PKcs were performed as described previously^30^.

### DNA annealing

DNA oligonucleotides (43 nucleotides) designed for blunt-end dsDNA were purchased from Sigma Aldrich. The complementary oligonucleotides were dissolved in ultrapure water to a final concentration of 100 μM, mixed at a molar ratio of 1:1 and annealed in a thermocycler. The mixed volume was first heated to 98 °C for 5 min and then cooled to 25 °C for 2 h to form a 43-base-pair (bp) dsDNA. The DNA sequences used for annealing can be found below.

dsDNA forward:

CCCGCTGCCGATTCCGCTGGAACATTAAAATTCGTCGACCTCC

dsDNA reverse:

GGAGGTCGACGAATTTTAATGTTCCAGCGGAATCGGCAGCGGG

### Sample preparation and cryo-EM data collection

Purified DNA-PKcs, Ku70/80 and 43-bp DNA were mixed at a molar ratio of 1.2:1:1 in 20 mM HEPES pH 7.6, 200 mM NaCl, 0.5 mM EDTA, 2 mM MgCl_2_, 2 mM DTT and 1 mM M3814 and incubated on ice for 1 h. After incubation, 3 μl of mixture was directly loaded to a Quantifoil R1.2/1.3 grid 300 Mesh Cu with graphene oxide (GO). Protein samples were left on a grid for 20 s, then blotted and plunge-frozen in liquid ethane using an FEI Vitrobot Mark IV system (Thermo Fisher Scientific) at 4 °C and 100% humidity. The grids were then clipped and transferred to a Titan Krios electron microscope operating at a voltage of 300 kV with a K3 direct electron detector (Gatan) at the cryo-EM facility of the Department of Biochemistry, University of Cambridge. Automated data collection was performed using the Thermo-Scientific EPU software package (Table 1).

### Image processing and model refinement

Data processing was done using Warp and cryoSPARC^31–33^. CTF correction, motion correction and particle auto-picking were carried out using Warp. All particles were subjected to ab initio reconstruction in cryoSPARC to generate four initial three-dimensional models. Classes of DNA-PKcs only and contamination were discarded and classes of DNA-PK were selected for further optimization through iterative rounds of heterogeneous refinement to filter out unwanted particles. The two models of DNA-PK in different states were then further refined using homogeneous refinement, global CTF refinement and nonuniform refinement in cryoSPARC. The reported resolutions were determined based on the ‘gold standard’ of the 0.143 Fourier shell correlation (FSC) criterion^34^. Maps were further improved using ResolveCryoEM in PHENIX^35^. Regarding the modeling of the complexes, DNA bases were manually built in COOT, and previously published cryo-EM structures of DNA-PK (PDB: 6ZHA, 7K1N, 7K0Y) were used as an initial reference for the protein modeling^13,14,36^. The templates were first rigid-body-fitted into the maps in CHIMERA and CHIMERAX, followed by real-space refinement in PHENIX^37–39^. The ligand was then modeled into the corresponding density in the cryo-EM maps in COOT, followed by multiple refinement rounds in PHENIX and model building in COOT^36,38^.

### Reporting summary

Further information on research design is available in the Nature Portfolio Reporting Summary linked to this article.

## Online content

Any methods, additional references, Nature Portfolio reporting summaries, source data, extended data, supplementary information, acknowledgements, peer review information; details of author contributions and competing interests; and statements of data and code availability are available at 10.1038/s41594-022-00881-w.

## Supplementary information

Reporting Summary

## Data Availability

Cryo-EM maps have been deposited in the Electron Microscopy Data Bank under accession numbers EMD-14546 (DNA-PK in the intermediate state) and EMD-14545 (DNA-PK in the active state). Model coordinates have been deposited in the Protein Data Bank under accession numbers 7Z88 (DNA-PK in the intermediate state) and 7Z87 (DNA-PK in the active state). Other structures used for modeling and comparison in this study were retrieved from the Protein Data Bank with accession codes 6ZFP for DNA-PKcs; 7OTY for the DNA-PKcs–inhibitor complex; and 6ZHA, 7K1N and 7K0Y for DNA-PK.
