# Supplementary material for: Human DNA-dependent protein kinase activation mechanism
Source: Nat Struct Mol Biol. 2023 Jan 5;30(2):140–7. doi: 10.1038/s41594-022-00881-w (PMC9935390; doi:10.1038/s41594-022-00881-w)
Supplement: Supplementary file 1 — Reporting Summary [file 41594_2022_881_MOESM1_ESM.pdf]

Corresponding author(s): Tom Blundell, Shikang Liang

Last updated by author(s): Oct 21, 2022

Reporting Summary

Nature Portfolio wishes to improve the reproducibility of the work that we publish. This form provides structure for consistency and transparency in reporting. For further information on Nature Portfolio policies, see our Editorial Policies and the Editorial Policy Checklist.

Statistics

For all statistical analyses, confirm that the following items are present in the figure legend, table legend, main text, or Methods section.

n/a	Confirmed	
☐	☒	The exact sample size (*n*) for each experimental group/condition, given as a discrete number and unit of measurement
☐	☒	A statement on whether measurements were taken from distinct samples or whether the same sample was measured repeatedly
☒	☐	The statistical test(s) used AND whether they are one- or two-sided<br>*Only common tests should be described solely by name; describe more complex techniques in the Methods section.*
☒	☐	A description of all covariates tested
☒	☐	A description of any assumptions or corrections, such as tests of normality and adjustment for multiple comparisons
☒	☐	A full description of the statistical parameters including central tendency (e.g. means) or other basic estimates (e.g. regression coefficient) AND variation (e.g. standard deviation) or associated estimates of uncertainty (e.g. confidence intervals)
☒	☐	For null hypothesis testing, the test statistic (e.g. *F*, *t*, *r*) with confidence intervals, effect sizes, degrees of freedom and *P* value noted<br>*Give P values as exact values whenever suitable.*
☒	☐	For Bayesian analysis, information on the choice of priors and Markov chain Monte Carlo settings
☒	☐	For hierarchical and complex designs, identification of the appropriate level for tests and full reporting of outcomes
☒	☐	Estimates of effect sizes (e.g. Cohen's *d*, Pearson's *r*), indicating how they were calculated

Our web collection on statistics for biologists contains articles on many of the points above.

Software and code

Policy information about availability of computer code

Data collection	Thermo-Scientific EPU 2.10.0
Data analysis	WARP v1.0.8, cryoSPARC v2.5.0, Phenix v1.19, COOT 0.8.9.2, Chimera 1.15.0, ChimeraX-1.1.1

For manuscripts utilizing custom algorithms or software that are central to the research but not yet described in published literature, software must be made available to editors and reviewers. We strongly encourage code deposition in a community repository (e.g. GitHub). See the Nature Portfolio guidelines for submitting code & software for further information.

Data

Policy information about availability of data

All manuscripts must include a data availability statement. This statement should provide the following information, where applicable:

- Accession codes, unique identifiers, or web links for publicly available datasets
- A description of any restrictions on data availability
- For clinical datasets or third party data, please ensure that the statement adheres to our policy

Cryo-EM maps have been deposited in the Electron Microscopy Data Bank under accession numbers EMD-14546 (DNA-PK in the intermediate state), EMD-14545 (DNA-PK in the active state). Model coordinates have been deposited in the Protein Data Bank under accession numbers 7Z88 (DNA-PK in the intermediate state), 7Z87 (DNA-PK in the active state).
Other structures used for modelling and comparison in this study were retrieved from the PDB with accession codes: 6ZFP for DNA-PKcs; 7OTY for DNA-PKcs/inhibitor complex; 6ZHA, 7K1N, 7K0Y for DNA-PK.

Field-specific reporting

Please select the one below that is the best fit for your research. If you are not sure, read the appropriate sections before making your selection.

☒ Life sciences ☐ Behavioural & social sciences ☐ Ecological, evolutionary & environmental sciences

For a reference copy of the document with all sections, see nature.com/documents/nr-reporting-summary-flat.pdf

Life sciences study design

All studies must disclose on these points even when the disclosure is negative.

Sample size	There were no statistical methods used to predetermine sample size. A total of 1094271 particles was collected for this study. The final particle numbers for reconstructions are comparable to recent cryo-EM studies in the field for the final reconstruction.
Data exclusions	During the cryo-EM data analysis, bad particles and contamination, including ice and protein aggregation, were discarded through iterative 3D classifications. This procedure is standard in the single particle analysis cryo-EM field.
Replication	Refinements of the DNA-PK structures were repeated at least twice with similar 3D maps.
Randomization	For cryo-EM, particles used in refinements were randomly split into two halves automatically and cross correlation coefficients between the two half sets are calculated.
Blinding	Particles were randomly split into two halves in cryo-EM data processing and resolution estimation. Blinding is not feasible for cryo-EM processing, because visual inspection is necessary to check the data quality.

Reporting for specific materials, systems and methods

We require information from authors about some types of materials, experimental systems and methods used in many studies. Here, indicate whether each material, system or method listed is relevant to your study. If you are not sure if a list item applies to your research, read the appropriate section before selecting a response.

Materials & experimental systems

n/a	Involved in the study	
☒	☐	Antibodies
☒	☐	Eukaryotic cell lines
☒	☐	Palaeontology and archaeology
☒	☐	Animals and other organisms
☒	☐	Human research participants
☒	☐	Clinical data
☒	☐	Dual use research of concern

Methods

n/a	Involved in the study	
☒	☐	ChIP-seq
☒	☐	Flow cytometry
☒	☐	MRI-based neuroimaging
